# Investigation of Chip Morphology in Elliptical Vibration Micro-Turning of Silk Fibroin

**DOI:** 10.3390/mi16010110

**Published:** 2025-01-19

**Authors:** Zhengjian Wang, Xichun Luo, Jining Sun, Wenkun Xie, Yinchuan Piao, Yonghang Jiang, Xiuyuan Chen

**Affiliations:** 1 Centre for Precision Manufacturing, DMEM, University of Strathclyde, Glasgow G1 1XJ, UK; zhengjian.wang@strath.ac.uk (Z.W.); w.xie@strath.ac.uk (W.X.); yinchuan.piao@strath.ac.uk (Y.P.); yonghang.jiang@strath.ac.uk (Y.J.); xiuyuan.chen@strath.ac.uk (X.C.); 2 School of Mechanical Engineering, Dalian University of Technology, Dalian 116023, China; jining.sun@dlut.edu.cn

**Keywords:** elliptical vibration micro-turning, smoothed particle hydrodynamics, chip morphology, silk fibroin, cutting chips, silk fibroin particles

## Abstract

Silk fibroin, known for its biocompatibility and biodegradability, holds significant promise for biomedical applications, particularly in drug delivery systems. The precise fabrication of silk fibroin particles, specifically those ranging from tens of nanometres to hundreds of microns, is critical for these uses. This study introduces elliptical vibration micro-turning as a method for producing silk fibroin particles in the form of cutting chips to serve as carriers for drug delivery systems. A hybrid finite element and smoothed particle hydrodynamics (FE-SPH) model was used to investigate how vibration parameters, such as frequency and amplitude, influence chip formation and morphology. This research is essential for determining the size and shape of silk fibroin particles, which are crucial for their effectiveness in drug delivery systems. The results demonstrate the superior capability of elliptical vibration micro-turning for producing shorter, spiral-shaped chips in the size range of tens of microns, in contrast to the long, continuous chips with zig-zag folds and segmented edges generated by conventional micro-turning. The unique zig-zag shapes result from the interplay between the high flexibility and hierarchical structure of silk fibroin and the controlled cutting environment provided by the diamond tool. Additionally, higher vibration frequencies and lower vertical amplitudes promote chip curling, facilitate breakage, and improve chip control, while reducing cutting forces. Experimental trials further validate the accuracy of the hybrid model. This study represents a significant advancement in the processing of silk fibroin film, offering a complementary approach to fabricating short, spiral-shaped silk fibroin particles with a high surface-area-to-volume ratio compared to traditional spheroids, which holds great potential for enhancing drug-loading efficiency in high-precision drug delivery systems.

## 1. Introduction

Silk fibroin, a natural protein extracted from the *Bombyx mori* silkworm, has recently garnered considerable interest due to its extraordinary mechanical characteristics [1] and beneficial biological attributes [2]. These unique properties have facilitated its use in various biomedical domains, including tissue engineering [3] and drug delivery [4]. In advanced drug delivery systems, the size and shape of particles are crucial for therapeutic applications, influencing biodistribution [5], cellular internalisation, and circulation times [6].

However, when manufacturing silk fibroin particles as carriers for drug delivery systems, traditional chemical-based techniques, such as electrospraying [7], desolvation [8] and salting out methods [9], face significant limitations, including high equipment costs [10,11] and environmental concerns associated with the use of hazardous chemicals [12,13]. To address these challenges, mechanical methods have emerged as promising alternatives for silk fibroin particle fabrication. Among these, Rajkhowa et al. [14] and Kazemimostaghim et al. [15] demonstrated that milling processes can produce silk spheroids that typically range in size from 10 to 100 microns. However, these traditional mechanical methods are often constrained by difficulties in maintaining shape integrity and size uniformity, as well as low production rates, limiting their broader applicability. Additionally, they are restricted to producing silk spheroids with a low surface-area-to-volume ratio and limited drug-loading capacity. In contrast, the elliptical vibration micro-turning process, an advanced mechanical technique, offers transformative potential in this field. By fabricating silk fibroin particles in the form of cutting chips with highly uniform and well-defined shapes and dimensions, this method ensures high production precision and efficiency and superior chip control, and effectively addresses the limitations of conventional approaches [16]. Furthermore, its capability to achieve precise control over particle size and shape underscores its suitability for applications that demand high-performance silk fibroin particles. In this process, chip morphology plays a crucial role in determining the size and shape of silk fibroin particles, which are vital for their effectiveness in drug delivery systems. Therefore, a comprehensive investigation of the chip morphology during the elliptical vibration micro-turning of silk fibroin is essential to obtain insights that can enhance particle design and optimise the particles’ application.

Previous research on vibration-assisted machining has shown that vibration parameters, such as amplitude and frequency, can significantly influence chip morphology. For instance, Muhammad et al. [17] investigated the machinability, chip formation, cutting forces, deformation zone temperature, and surface roughness in traditional and ultrasonic-assisted titanium alloy turning. The ultrasonic-assisted process demonstrated superior machinability, characterised by lower cutting forces, improved surface roughness, and shorter chips compared to the conventional method. Moreover, cutting parameters play a crucial role in the cutting process [18,19]. For example, optimal results for producing discrete chips can be achieved by carefully selecting parameters such as cutting speed and vibration amplitude. Chen et al. [20] developed guidelines for predicting chip morphology during the ultrasonic vibration drilling of stainless steel. They observed that fragmented chips form only when the amplitude-to-frequency ratio meets specific criteria. Dambatta et al. [21] studied ultrasonic-assisted grinding for machining metallic materials and found that incorporating ultrasonic vibration enhances material removal rates and surface integrity. They also discovered that vibration parameters, such as amplitude and frequency, have a substantial impact on the residual stress within the workpiece. Li, Wu, and Nomura [22] observed that the type of chips produced during the grinding of Inconel 718 (i.e., shear chip, knife chip and flow chip) depends significantly on the amplitude of the tool vibration. Yang et al. [23] highlighted that in vibration-assisted drilling of Ti-6Al-4V titanium alloys, using a low rotational speed combined with a high vibration frequency can promote a shift in chip morphology from continuous to discontinuous. Li et al. [24] obtained discontinuous copper chips through vibration-assisted cutting. Their research identified tool vibration amplitude and depth of cut as key parameters influencing chip morphology and breakage, underscoring the importance of the precise adjustment of vibration parameters for effective chip control.

Although the effects of tool vibration on the chip morphology of metallic materials have been well studied, the elliptical vibration micro-turning process of silk fibroin has not been extensively explored, particularly regarding the impact of vibration parameters such as amplitude and frequency. This presents a significant knowledge gap. Analysing the chip morphology generated during this process can provide valuable insights into the fundamental material removal mechanisms. Understanding these mechanisms at the microscale is crucial for optimising the production of silk fibroin particles with the desired chip morphology and precise dimensions. Additionally, unlike metallic materials, silk fibroin possesses a complex molecular structure composed of a random amorphous matrix and beta-sheet crystallites [25]. Its mechanical characteristics, including Young’s modulus, hardness, toughness, and yield strength, are distinct from those of metals such as copper, steel, Inconel, and titanium alloys. Table 1 highlights the significant mechanical differences, such as the considerably lower Young’s modulus and hardness of silk fibroin, which strongly affect its material removal mechanisms and chip morphology during elliptical vibration micro-turning, presenting a substantial challenge for this research.

This study aims to address the knowledge gap by systematically investigating how vibration parameters, such as amplitude and frequency, affect chip morphology in the elliptical vibration micro-turning of silk fibroin. Numerical simulations provide an in-depth understanding of the cutting process that is difficult to observe experimentally, making them a crucial complement to experimental methods. To achieve this, we will employ a hybrid FE-SPH approach, integrating the merits of smoothed particle hydrodynamics (SPH) and the finite element method (FEM) [31,32]. This hybrid formulation aims to deliver detailed insights into the dynamics of chip formation and chip morphology. The findings have significant potential for enhancing the dimensional quality of fabricated silk fibroin particles.

The structure of this paper is organised to present the research findings comprehensively. Section 2 introduces the development of a hybrid FE-SPH orthogonal cutting model for simulating the chip formation process in elliptical vibration micro-turning of silk fibroin. Section 3 explores the effects of vibration parameters, specifically frequency and amplitude, on chip morphology, cutting force, and residual stress through detailed numerical simulations. Section 4 validates the simulation results with experimental machining trials, demonstrating the accuracy of the numerical model.

## 2. Hybrid FE-SPH Modelling

### 2.1. Elliptical Vibration Micro-Turning Process

**Figure 1:**
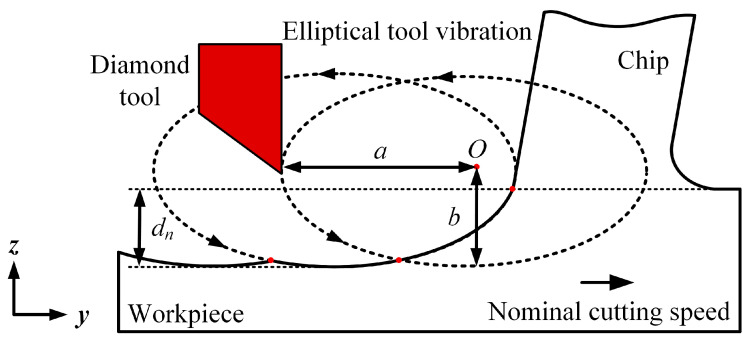
Elliptical vibration micro-turning process.

Figure 1 provides a schematic illustration of the elliptical vibration micro-turning process, which is assumed to follow an orthogonal cutting scheme [33,34]. This assumption is necessary and effective as it dramatically reduces the computational power needed while maintaining high modelling accuracy [35].

In the numerical model for elliptical vibration micro-turning, the diamond tool undergoes periodic oscillations in both the cutting direction (*y*-axis) and the depth of cut direction (*z*-axis), creating elliptical tool paths. In a typical diamond turning machine, as adopted by the authors in this work, the *x*-axis represents the radial direction (i.e., the feed direction), which corresponds to the movement of the diamond tool perpendicular to the axis of rotation of the workpiece. The phase angle between these two oscillations is consistently set at 90°. The following equations can express the displacements of the diamond tool in the *y* and *z* directions:

(1) $$\left\{\begin{array}{c} y\left(t\right)=acos\left(2\pi ft\right)-V_{w}t-a\\ z\left(t\right)=-bsin(2\pi ft) \end{array}\right.$$

where *a* and *b* are the amplitudes of vibration in the horizontal and vertical directions, respectively, and *f* is the vibration frequency. The nominal cutting speed of the workpiece is denoted as *V_w_*. The nominal depth of cut, *d_n_*, is defined as the vertical distance from the surface of the workpiece to the maximum depth reached by the diamond tool. It is worth noting that in conventional micro-turning, the nominal depth of cut is equivalent to the actual depth of cut.

### 2.2. Orthogonal Cutting Model

**Figure 2:**
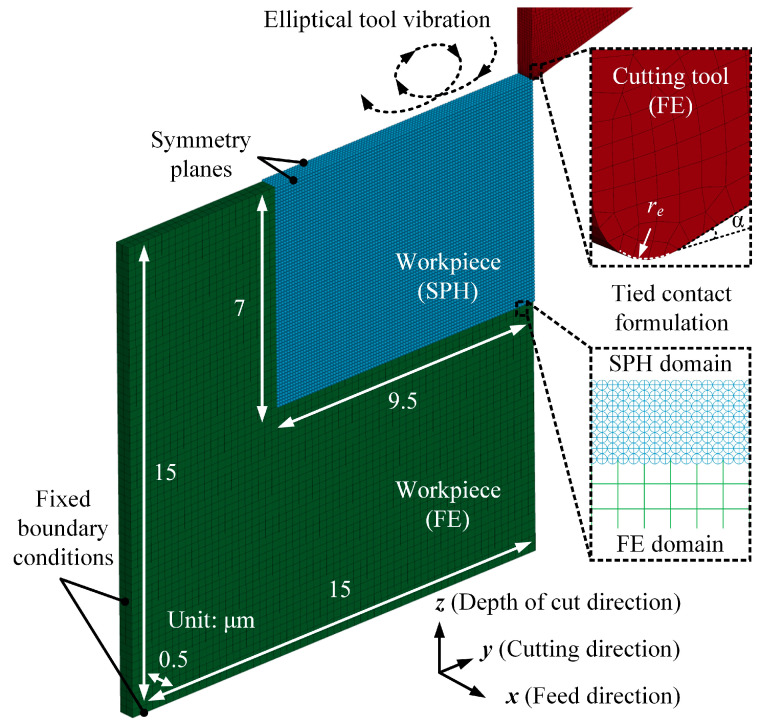
Hybrid FE-SPH numerical model for elliptical vibration micro-turning.

A three-dimensional workpiece, measuring 15 μm by 15 μm, with a thickness of 0.5 μm, was created with the aid of LS-PrePost. Thermal effects in this process are minimal and considered negligible [36] because the tool and workpiece engage and separate periodically, allowing for effective cooling during each cycle, which dissipates heat and ensures the stability of silk fibroin [16,37]. Additionally, the use of shallow cuts, approximately 1 μm in depth, and the consequent low material removal rates further mitigate thermal impacts [38]. Machining trials were also performed at low speeds to minimise heat generation. The hybrid FE-SPH numerical model developed in this study is presented in Figure 2.

In this model, the workpiece is divided into two distinct domains: an SPH domain, measuring 9.5 μm × 7 μm × 0.5 μm, designed to balance computational efficiency and accuracy using the renormalised formulation; and a remaining FE domain. The FE region is constructed using Lagrangian elements with a mesh size of 0.25 μm, and the SPH domain is created with a particle density of 0.1 μm. Sensitivity analyses were performed to confirm the appropriate mesh size and particle density. Two symmetry-plane constraints are applied to the front and back surfaces of the SPH domain to generate ghost particles along the feed direction (*x*-axis), ensuring kernel compactness [39]. Fixed boundary conditions are imposed on the left and bottom surfaces of the FE region of the workpiece. A tied contact formulation is employed at the interface between the FE and SPH domains using *CONTACT_TIED_NODES_TO_SURFACE via the graphical user interface within LS-PrePost, whose effectiveness has been validated in prior research [40]. The tool is defined by a rake angle of 0°, a clearance angle (*α*) of 15°, and an edge radius (*r_e_*) of 60 nm. The mesh surrounding the tool tip has been refined sufficiently to enhance model accuracy. Silk fibroin is a protein with relatively low mechanical hardness, and its interaction with a smooth diamond surface naturally results in a low friction coefficient, so the Coulomb friction coefficient between the diamond tool and the silk fibroin workpiece was set at 0.12 [41]. The elastoplastic material model, incorporating isotropic/kinematic hardening plasticity, was used to represent the mechanical behaviour of silk fibroin, while its damage behaviour was characterised using the Cowper–Symonds material model:

(2) $$\left\{\begin{array}{c} \sigma_{d}=\left[1+{\left(\frac{\dot{\varepsilon_{d}}}{D}\right)}^{1/p}\right](\sigma_{s}+\beta E_{p}\varepsilon_{d})\\ E_{p}=E_{TAN}E/\left(E-E_{TAN}\right) \end{array}\right.$$

where *σ_d_* is the dynamic yield stress and *σ_s_* is the static yield strength. *ε_d_* and $\dot{\varepsilon }$*_d_* are the dynamic strain and strain rate, respectively. *E* is the Young’s modulus. *E_p_* represents the plastic hardening modulus, and *E_TAN_* is the tangent modulus, describing the slope of the stress–strain curve during the plastic phase. *D* and *p* are a set of material parameters. Parameter *p* is dimensionless, while parameter *D* has the same unit as strain rate. These parameters have been thoroughly discussed in the authors’ previous work [42]. Parameter *β* is the material hardening parameter, which can be used to manipulate isotropic hardening, kinematic hardening, or a mix of isotropic and kinematic hardening. In this study, *β* is set to 0 [43], reflecting the significant shear deformation observed in the actual cutting process. A detailed summary of the material parameters for both the diamond tool and the silk fibroin workpiece is provided in Table 2. It is worth noting that the accuracy of this hybrid model in predicting specific cutting forces and chip morphology has been validated in previous studies [42,44].

**Figure 3:**
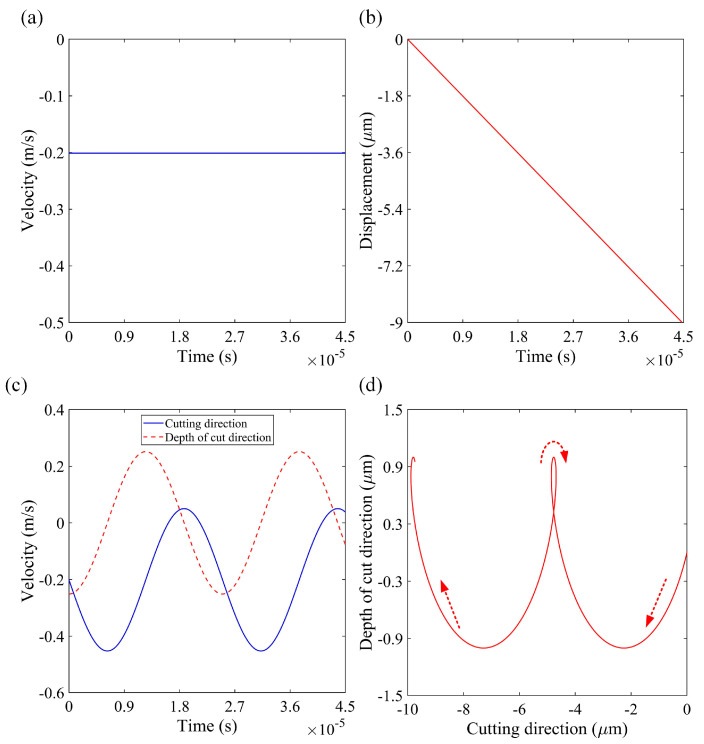
Typical variations in tool velocity and displacement: (**a**) velocity in conventional micro-turning; (**b**) displacement in conventional micro-turning; (**c**) velocity in elliptical vibration micro-turning; (**d**) displacement in elliptical vibration micro-turning.

One of the most critical boundary conditions in this model is the variation in the velocity and displacement of the cutting tool. Figure 3 illustrates these typical variations for conventional micro-turning and elliptical vibration micro-turning. For comparability, the same nominal cutting speed of 0.2 m/s was employed in all cases; this speed was selected solely for demonstration purposes. The vibration amplitudes of the tool in the cutting and depth of cut directions were 2 μm, with a frequency of 40 kHz. The linear and elliptical motions of the cutting tool were incorporated as boundary conditions in the orthogonal model. The primary difference between conventional micro-turning and elliptical vibration micro-turning is the tool/workpiece engagement. Conventional micro-turning involves continuous contact, while elliptical vibration micro-turning has intermittent engagement. In terms of chip morphology, which is the focus of this work, elliptical vibration micro-turning produces smaller, more uniform chips with improved chip control capacity due to its discontinuous cutting action.

## 3. Parametric Study

### 3.1. Effect of Vibration Frequency

**Figure 4:**
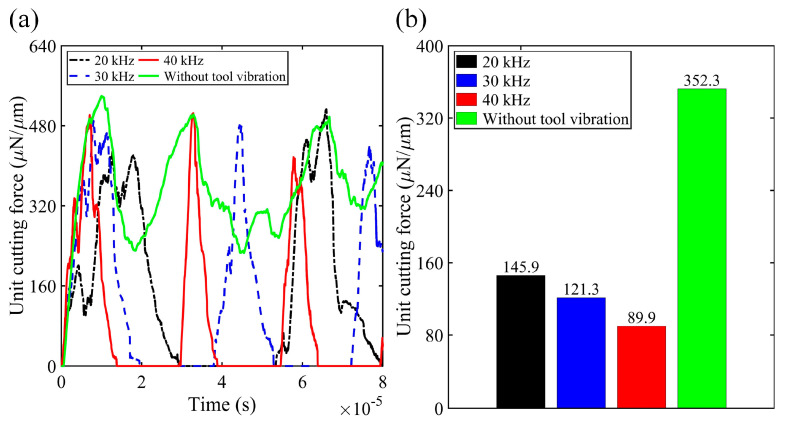
(**a**) Time-domain signatures of the unit cutting force during conventional micro-turning (green curve, without tool vibration) and elliptical vibration micro-turning at different vibration frequencies (20 kHz, 30 kHz, and 40 kHz). (**b**) Comparison of the average unit cutting force for each condition, showing a significant reduction in cutting force as the vibration frequency increases.

In this subsection, considering the constraints of available computational resources, three frequency levels (20 kHz, 30 kHz, and 40 kHz) were adopted in the simulation studies to investigate the effect of vibration frequency on the elliptical vibration micro-turning of silk fibroin. The vibration amplitudes *a* and *b* were set to 1 μm and kept constant throughout the simulations. Cutting simulations were conducted under the same nominal cutting speed for conventional and elliptical vibration micro-turning processes. Specifically, the nominal depth of cut in both processes was set equal to the vertical vibration amplitude *b* at 1 μm. Therefore, the simulation results of conventional micro-turning can be used as a benchmark for the elliptical vibration process. Notably, despite the use of different frequencies in the cases, all simulations were run for the same duration to ensure better demonstration. Additionally, the simulated cutting forces were normalised in the *x*-direction for ease of comparison. Figure 4 shows the signatures and their average values of the normalised unit cutting force.

In this subsection, considering the constraints of available computational resources, three frequency levels (20 kHz, 30 kHz, and 40 kHz) were adopted in the simulation studies to investigate the effect of vibration frequency on the elliptical vibration micro-turning of silk fibroin. The vibration amplitudes *a* and *b* were set to 1 μm and kept constant throughout the simulations. Cutting simulations were conducted under the same nominal cutting speed for conventional and elliptical vibration micro-turning processes. Specifically, the nominal depth of cut in both processes was set equal to the vertical vibration amplitude *b* at 1 μm. Therefore, the simulation results of conventional micro-turning can be used as a benchmark for the elliptical vibration process. Notably, despite the use of different frequencies in the cases, all the simulations were run for the same duration (i.e., 8 × 10^−5^ s) to ensure consistency in demonstration. Additionally, the simulated cutting forces were normalised as the unit cutting force, calculated by dividing the simulated cutting force by the model dimension in the *x*-direction, for ease of comparison. Figure 4 shows the signatures and their average values of the normalised unit cutting force. Please be aware the bar diagrams on the right-hand side are not intended to show how the cutting force varies over time; instead, they summarise the average force for each case.

As shown in Figure 4, the peak values of the unit cutting force remained nearly constant across all simulation cases. However, the average values continuously decreased as the frequency increased from 20 kHz to 40 kHz. At 20 kHz, there was a 58.6% reduction in unit cutting force compared to conventional micro-turning. Increasing the vibration frequency to 30 kHz and 40 kHz further reduced the average cutting force by 65.6% and 74.5%, respectively. The reduction in cutting force with increasing vibration frequency is a result of the intermittent cutting mechanism induced by elliptical vibration. At higher frequencies, the tool spends less time in contact with the workpiece during each oscillation cycle, which lowers the average cutting force.

**Figure 5:**
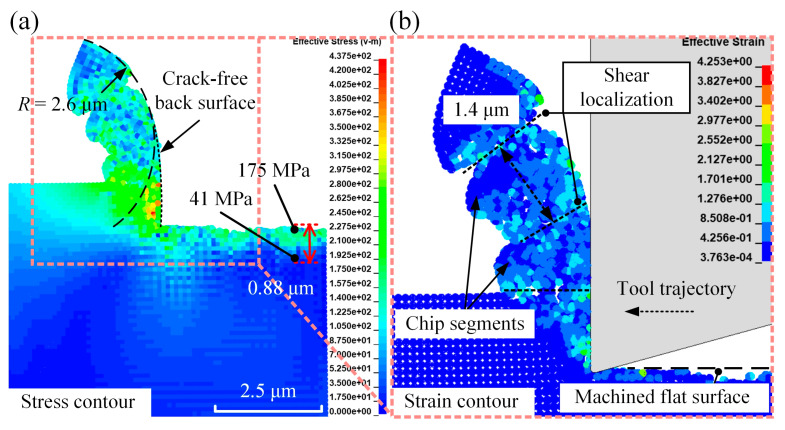
The distribution of (**a**) von Mises stress (in MPa) and (**b**) effective plastic strain in conventional micro-turning. The right-hand side of the panel provides an enlarged view of the left side.

**Figure 6:**
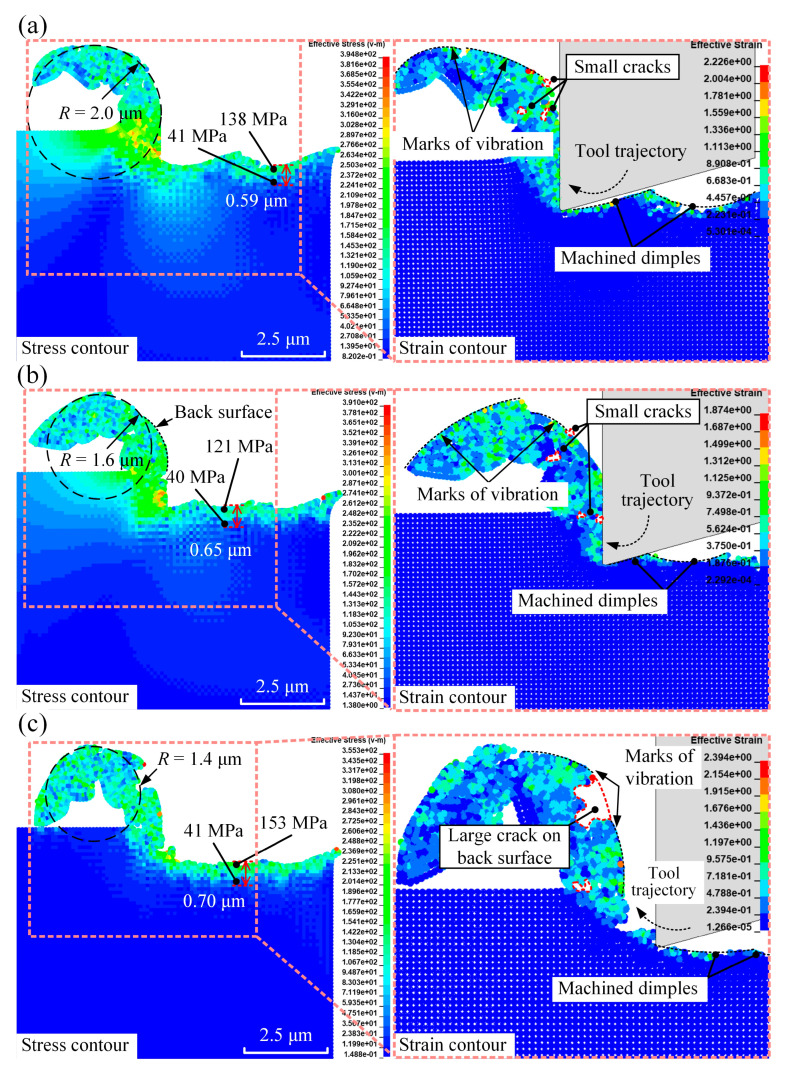
The distribution of von Mises stress in elliptical vibration micro-turning, with a fixed vibration amplitude of 1 μm, at different frequencies: (**a**) 20 kHz, (**b**) 30 kHz, and (**c**) 40 kHz. Enlarged views are provided on the right-hand side of each panel.

Figure 5 presents the distribution of von Mises stress and effective plastic strain for conventional micro-turning. Figure 5b illustrates that chip segments resulting from shear localisation were formed on the simulated cutting chip. This observation indicates that the shear localisation mechanism, with a segmentation pitch of approximately 1.4 μm, has been successfully reproduced in the simulation. Moreover, due to the stable and continuous cutting effect of the diamond tool in the conventional process, a cutting chip with a smooth, crack-free back surface and a flat surface machined on the silk fibroin workpiece can be identified from Figure 5. For comparison, Figure 6 illustrates the von Mises stress distribution for elliptical vibration micro-turning at various frequencies.

Across all the figure panels in Figure 6, the dimple structures left on the machined workpiece surface are due to the elliptical trajectory of the diamond tool, which replicated this pattern onto the workpiece. Regarding the dimples and their radius of curvature, this study focuses on chip morphology rather than surface dimples. Consequently, an analysis of the dimples’ radius or their variations in size has not been conducted, as it falls outside the scope of this work. Compared to conventional micro-turning, this elliptical trajectory also reduced the unit cutting force, leading to a decrease in residual stress on the machined surface. The maximum residual stress and the depth of the residual stress field were both greater in conventional micro-turning than in elliptical vibration micro-turning. Additionally, as shown in Figure 6, an increase in vibration frequency resulted in higher maximum residual stress and a deeper residual stress field due to the larger number of tool impacts on the workpiece per unit time.

**Figure 7:**
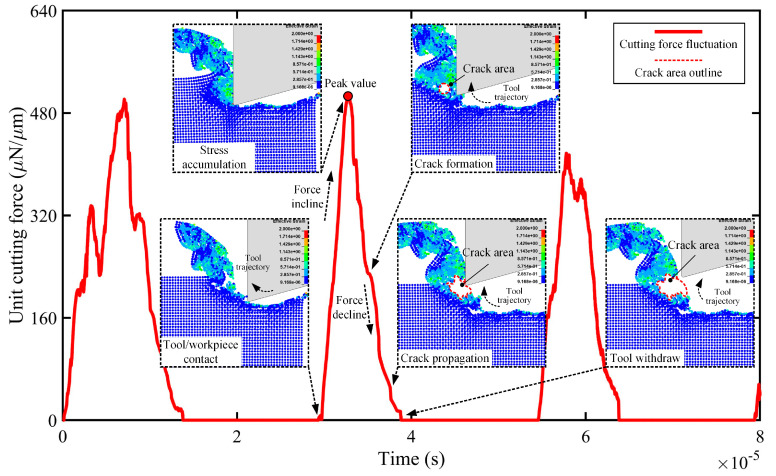
The simulated unit cutting force (unit: μN/μm) oscillation with a vibration amplitude of 1 μm and a frequency of 40 kHz.

Moreover, the chip morphologies, particularly the chip radius of curvature *R*, differed significantly between conventional and elliptical vibration micro-turning. As illustrated in Figure 5a, the simulated radius of curvature for conventional micro-turning was 2.6 μm, which was higher than those observed with elliptical vibration. Figure 6 indicates that increasing the frequency further reduced the radius of curvature from 2.0 μm to 1.4 μm. This change was attributed to the elliptical vibro-impact of the diamond tool, which concentrated more energy within the cutting zone and increased the degree of chip curling. Furthermore, only small cracks within the cutting chips were observed in Figure 6a,b. In contrast, as shown in Figure 6c, a much larger crack on the back surface of the cutting chip with marks of vibration can be identified in the simulation case with a frequency of 40 kHz, and its formation process is illustrated in Figure 7.

Figure 7 illustrates the strain distributions within the cutting chip during the second cutting cycle and the oscillation of the simulated unit cutting force. Once the diamond tool engaged the silk fibroin workpiece, the unit cutting force steadily increased until the tool reached the lowest point in its trajectory, after which the force gradually diminished to zero. As the cutting tool advanced, stress accumulated in front of the tool tip, eventually leading to crack formation in the cutting chip when the stress intensity factor exceeded the fracture toughness of the silk fibroin film. Consequently, the stress was released, evidenced by a rapid decline in the unit cutting force.

The formation and propagation of cracks during this process align with Griffith’s criterion [45]. During elliptical vibration micro-turning, the periodic motion of the tool generates alternating stress fields, increasing the energy release rate and facilitating crack initiation at critical points. Once initiated, these cracks propagate along the tool trajectory under the combined effects of tensile stress and the tool’s shearing action. This interplay between stress accumulation, crack initiation, and propagation provides valuable insights into the material removal mechanism.

After reaching its peak value, the cutting force decreased significantly. This reduction in force was due to crack formation within the cutting chip, which became more evident as the volume of removed material decreased. Furthermore, under the combined effect of the diamond tool’s elliptical trajectory and its shearing action on the silk fibroin workpiece, the tool tip stretched the crack, causing it to propagate and expand. At a higher vibration frequency, such as 40 kHz, the process completed in a shorter time (approximately 50% shorter than at 20 kHz), with the subsequent higher energy density further increasing crack expansion, resulting in a larger crack on the back surface of the chip compared to at lower frequencies.

The strain distribution also varied across different cutting cycles. During the first cutting cycle, strain progressively accumulated, leading to the formation of cracks and subsequent material removal. However, in the third cutting cycle, the strain intensities were notably lower, as indicated by the reduced peak cutting force. This reduction is likely due to the altered deformation behaviour of the material after repeated cyclic loading, which may involve stress relaxation or changes in fracture energy. These findings demonstrate the dynamic nature of strain evolution during elliptical vibration micro-turning and its impact on the material removal mechanism.

These findings suggest that, compared to the conventional process, the additional vibrations in elliptical vibration micro-turning significantly reduce the chip radius of curvature, transforming the chip morphology from flat to spiral with noticeable curling. Moreover, higher vibration frequencies promote the formation of larger cracks on the back surface, enhancing chip breakage and resulting in finer particles. According to fracture mechanics, crack growth occurs when the energy release rate exceeds the material’s critical fracture energy, as outlined by Griffith’s criterion. At higher frequencies, the cutting tool oscillates more rapidly, creating alternating stress fields with greater intensity. These intensified stress fields increase the concentration of tensile stress at the crack tips, accelerating both crack initiation and propagation. As a result, the larger cracks formed at higher frequencies contribute to more efficient chip breakage and the production of finer particles.

### 3.2. Effect of Vibration Amplitude

Four levels of vertical vibration amplitude *b* (0.5 μm, 0.75 μm, 1 μm, and 1.5 μm) were selected to analyse the effect of vibration amplitude on elliptical vibration micro-turning. The nominal depths of cut were set to be equal to the vertical vibration amplitude in all simulation cases. All the simulations were conducted at a vibration frequency of 20 kHz, with a nominal cutting speed of 0.05 m/s, for consistent comparison.

**Figure 8:**
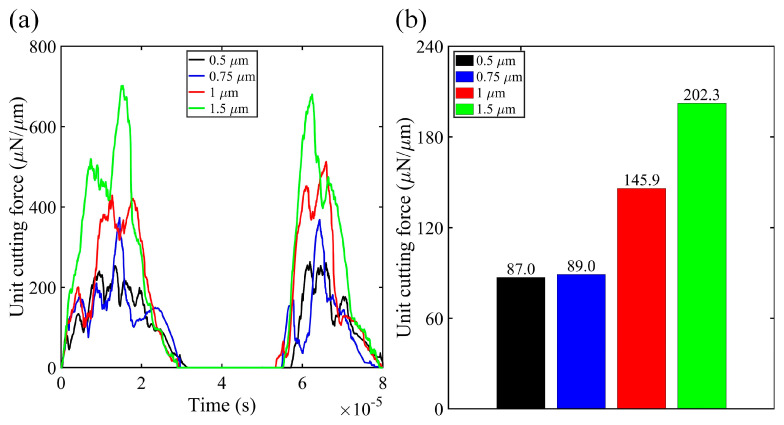
(**a**) Time-domain signatures of the unit cutting force for elliptical vibration micro-turning at various vibration amplitudes (0.5 µm, 0.75 µm, 1 µm, and 1.5 µm), showing how the cutting force varies with time during the machining process. (**b**) Average unit cutting force values for each vibration amplitude, highlighting the relationship between increased vibration amplitude and cutting force.

As shown in Figure 8, the increase in vibration amplitude led to a rise in the unit cutting force. This rise was due to the greater nominal depth of cut, resulting in a larger volume of silk fibroin being removed.

**Figure 9:**
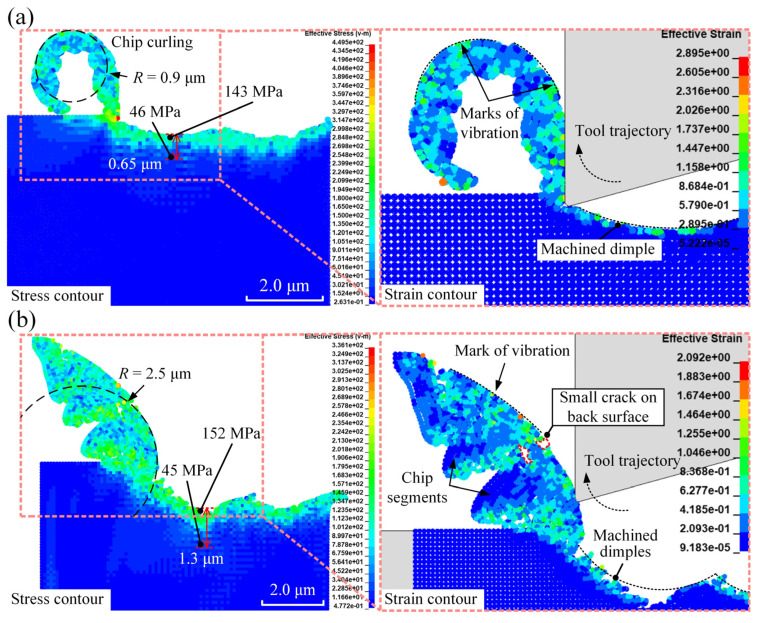
The distribution of effective plastic strain and von Mises stress (unit: MPa) in elliptical vibration micro-turning with a fixed vibration frequency of 20 kHz, at different amplitudes of (**a**) 0.5 μm and (**b**) 1.5 μm. The left-hand side illustrates the stress contour, highlighting key features such as chip curling and the residual stress field. While the overall stress variation is similar, the depth of the residual stress field differs significantly between (**a**,**b**). The right-hand side provides an enlarged view to better demonstrate the strain contour.

Figure 9 illustrates the chip morphology for elliptical vibration micro-turning with a fixed vibration frequency of 20 kHz, at different amplitudes.

For the case with a vibration amplitude of 1.5 µm, the chip segments and shear localisation were more pronounced compared to the case with a vibration amplitude of 0.5 µm. This is because a larger nominal depth of cut increased the spacing between shear bands, making the chip segments more visible. This observation aligns well with expectations based on a previous study [46]. To maintain clarity and focus, only the extreme cases of vibration amplitudes (0.5 µm and 1.5 µm) are included in the analysis, as they effectively illustrate the significant differences in chip morphology and residual stress behaviour without overcomplicating the visual representation.

The simulations demonstrated that vibration amplitude significantly impacts chip morphology, which, in turn, influences chip breakage in elliptical vibration micro-turning. An increase in amplitude raised the chip radius of curvature *R* from 0.9 µm to 2.5 µm due to greater chip thickness and stiffness. Additionally, higher residual stress and a deeper residual stress field were observed on the machined surface with greater vibration amplitude. This occurred because the diamond tool wedged deeper into the silk fibroin, leading to increased deformation and effective stress, thereby raising the residual stress in the machined surface.

In summary, the simulated results provide valuable insights into the material removal mechanisms and chip formation during the elliptical vibration micro-turning of silk fibroin. By analysing the cutting parameters and chip morphology, this study demonstrates the process’s capability to produce smaller, uniform fibroin particles, which can serve as carriers for drug delivery systems. The discontinuous cutting action inherent in elliptical vibration micro-turning leads to improved chip control and enhanced chip breakage, resulting in the generation of fine particles. These findings suggest that optimising the process parameters, such as vibration amplitude and frequency, could further enhance particle size control, making the technique promising for applications in drug delivery systems.

## 4. Experimental Validation

### 4.1. Experimental Setup and Conditions

**Figure 10:**
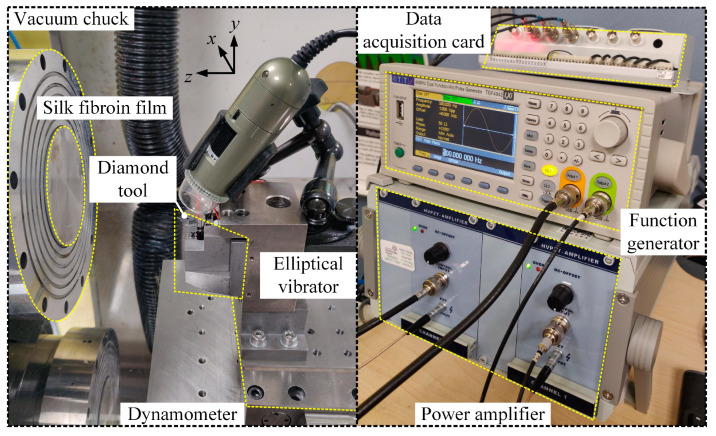
Experimental setup: the left panel shows the machining configuration, and the right panel depicts the signal generation and data acquisition setup.

Before conducting the micro-turning experiments, the silk fibroin solution extracted from *Bombyx mori* cocoons was cast onto an aluminium plate to form a silk fibroin film with a thickness of approximately 0.5 mm. This ensured that the cutting process predominantly affected the silk fibroin layer, without interference from the aluminium base. The cutting operations employed a 0.5 mm nose radius, a 0° rake angle, and a 15° clearance angle. As shown in Figure 10, a high-resolution dynamometer (9129AA, Kistler, Winterthur, Switzerland) was employed to monitor the cutting forces. A Hitachi S3700-N (Tokyo, Japan) scanning electron microscope (SEM) was also utilised to observe and analyse the silk cutting chips.

Accurate sinusoidal signals were generated using a two-channel signal generator (TGF4042, Aim-TTi, Huntingdon, UK) and a high-end power amplifier (E01.A2, CoreMorrow, Harbin, China). These signals were crucial for ensuring the proper frequencies and phase alignment necessary to coordinate the oscillations of the cutting tool in the *y*- and *z*-directions. Structural reliability of the system was achieved using solid low-voltage piezoelectric actuators (PSt150, Piezomechanik, Munich, Germany) and a well-designed elliptical vibrator. This setup adequately mitigated asynchronous movements between two directions, as confirmed in previous research by the authors [47]. The system was rigorously calibrated before operation to ensure the actuators responded precisely to the control signals.

Table 3 lists the processing parameters for four machining trial sets: I, II, III, and IV. The experiments employed two vibration frequencies: 100 Hz and 500 Hz. All the experiments were conducted at a nominal cutting speed of 0.42 mm/s (along *y*-axis), with a uniform feed rate of 150 μm/rev (along *x*-axis), to ensure non-overlapping cutting.

### 4.2. Results and Discussion

**Figure 11:**
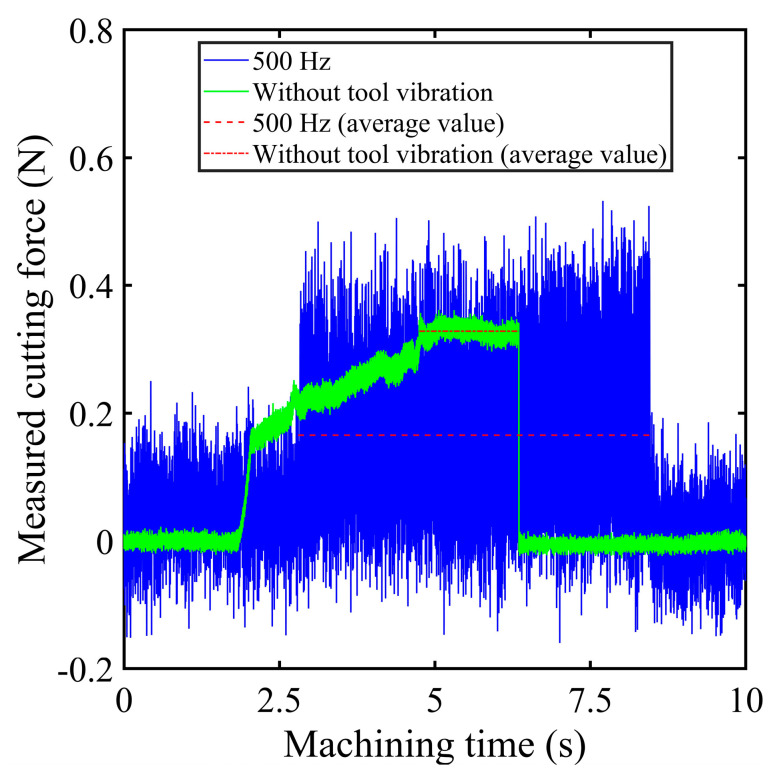
Comparison of cutting force variations between conventional micro-turning (*d_n_* = 1 μm) and elliptical vibration micro-turning (*f* = 500 Hz; *d_n_* = 1 μm). The blue and green curves represent the measured cutting forces for the vibration-assisted and non-vibration conditions, respectively, while the dashed lines indicate their average values.

Figure 11 compares the cutting forces from the elliptical vibration micro-turning experiment (set III) with those from the conventional micro-turning process (set I).

Figure 11 reveals that, in elliptical vibration micro-turning, the peak cutting force is marginally greater than in traditional micro-turning. This increase is due to the vibration impact of the diamond tool. However, the cutting force drops to almost zero at the lowest points of each vibration cycle, showing that the tool and workpiece were fully separated. The hybrid model proposed in this study effectively captures the periodic variations in cutting force in this process, demonstrating its effectiveness in replicating the actual machining conditions. The failure to achieve an absolute zero value during the acquisition process resulted in the signal noise from the force data. In vibration cutting, the average cutting force decreases significantly from 0.33 N to 0.17 N, which represents a 48.5% reduction compared to conventional micro-turning. This reduction is due to the intermittent contact between the tool and workpiece, achieved by applying elliptical vibration [48,49].

**Figure 12:**
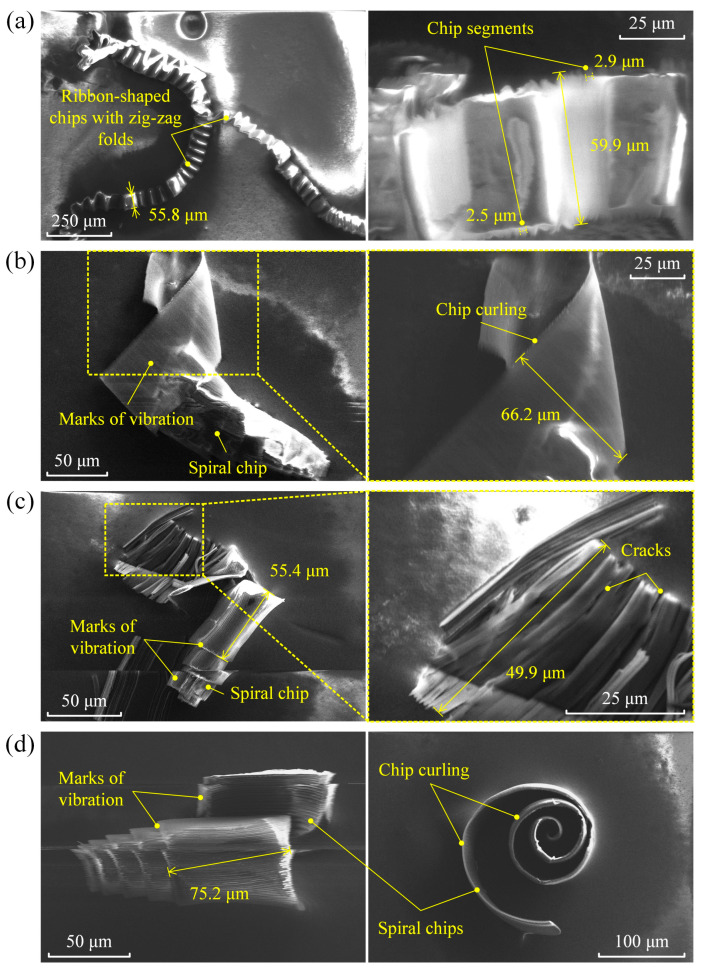
SEM images of the chip morphologies obtained in the experiments of (**a**) set I, (**b**) set II, (**c**) set III, and (**d**) set IV.

Figure 12 presents a series of SEM images showing the chip morphologies from the experiments conducted in sets I, II, III, and IV.

In Figure 12a, it can be seen that the conventional micro-turning process produced long, continuous, ribbon-shaped chips with zig-zag folds and segmented edges. The developed hybrid numerical model successfully replicated the formation of chip segments with a pitch of approximately 2.7 μm, despite this being double the simulated value of 1.4 μm, as illustrated in Figure 5b. The variation in the predicted chip segmentation arose from thermal softening not being fully accounted for, which led to a smaller predicted pitch compared to the actual result. In the experiment of set I, under the shearing effect of the diamond tool, the chip curling speed was lower than the chip generation speed. This discrepancy caused the chips to undergo significant deformation under the squeezing action of the cutting tool, leading to the formation of zig-zag folds. The zig-zag pattern suggests a buckling phenomenon, where the chip material, under compressive stresses, folds into a periodic structure. This phenomenon is more pronounced when the material being removed is thin and flexible. Specifically, the Young’s modulus of silk fibroin is only 5.2 GPa; in this case, the depth of cut was just 1 μm. The hierarchical structure of silk fibroin, with its mixture of crystalline and amorphous regions, also contributes to these non-uniform deformation patterns.

In contrast, metals generally form continuous spiral chips due to their homogeneous structure and high shear strength, which allow for smooth, consistent plastic deformation. With the introduction of elliptical vibration, the chip morphology changed from long and ribbon-shaped, to short and spiral-shaped with significant curling. Vibration marks were clearly visible throughout the silk chips, as shown in Figure 12b–d. This transition in chip morphology was due to the additional vibration of the diamond tool compared to the conventional process. As discussed in Section 3, the tool vibration significantly decreased the chips’ radius of curvature, making the chips more prone to curling. Figure 12c shows cracks within the chips, which facilitated chip breakage and resulted in short, spiral-shaped chips.

The three-dimensional dimensions of the spiral-shaped chips, as illustrated in Figure 12, were measured to evaluate their size and potential applications. The chip width ranged from approximately 49.9 μm to 75.2 μm, while the spiral diameter was about 150 μm, depending on the experimental conditions. These dimensions highlight the ability of elliptical vibration micro-turning to produce consistent and controlled chip sizes, which are advantageous for biomedical applications. The spiral-shaped chips exhibit a high surface-area-to-volume ratio, enabling efficient interaction with therapeutic agents and improving drug encapsulation and release kinetics. Compared to traditional ribbon-shaped chips, the spiral-shaped chips are more compact and have regular structures, making them suitable for targeted drug delivery applications.

The ribbon-shaped chips also hold potential for biomedical applications, due to their high surface-area-to-volume ratio compared to traditional spheroid particles. This unique property could enhance drug-loading efficiency in specific high-capacity drug delivery scenarios. The zig-zag folds in the ribbon-shaped chips, caused by compressive stresses and the buckling phenomenon, further increase the available surface area for drug interaction.

Together, both ribbon-shaped and spiral-shaped chips offer unique properties that could be utilised in drug delivery systems. While the spiral-shaped chips are ideal for precise and controlled drug delivery applications, the ribbon-shaped chips may be beneficial for scenarios requiring higher drug-loading capacities. Future research will focus on evaluating the drug-loading efficiency, release kinetics, and therapeutic performance of these silk fibroin chips for biomedical applications.

In summary, lower vertical vibration amplitudes, combined with smaller nominal depths of cut, reduce the chips’ radius of curvature. Additionally, higher frequencies further decrease the chips’ radius of curvature, and are likely to induce crack initiation and propagation on the back surface of the chips. These factors significantly promote chip breakage, highlighting the superior chip-breaking capability of elliptical vibration micro-turning compared to the conventional process. It is worth mentioning that the difference in speed was due to the differing frequencies used in the simulations (100 and 500 Hz) compared with the experiments (20, 30, and 40 kHz), which was necessitated by practical constraints. Performing simulations at the actual experimental frequencies would have significantly increased computational demands, rendering the work impractical given the limitations of available computing power. Despite these differences, both the simulations and experiments demonstrated similar trends in chip morphology transitions. Future work will focus on refining and optimising computational methods to simulate at experimental frequencies. After this, in vitro experiments will be carried out to validate the drug delivery effectiveness of the silk fibroin particles fabricated using our proposed vibration micro-turning method.

## 5. Conclusions

This research explored the effect of vibration parameters on chip morphology in the elliptical vibration micro-turning of silk fibroin, using a hybrid FE-SPH model validated through experimental trials. The results demonstrate that elliptical vibration micro-turning significantly modifies chip morphology, transforming long, ribbon-shaped chips into short, spiral-shaped chips with high consistency and controllability. Specifically, higher vibration frequencies and lower vertical amplitudes promote chip curling, facilitate chip breakage through induced surface cracks, and improve overall chip control. These findings highlight the superior chip-breaking capability of elliptical vibration micro-turning compared to conventional methods, making it an effective technique for producing spiral-shaped silk fibroin particles.

The study also shows that elliptical vibration micro-turning enables the precise fabrication of silk fibroin particles with dimensions ranging from tens to hundreds of microns, which is critical for their application as carriers in drug delivery systems. The spiral-shaped chips produced exhibit a high surface-area-to-volume ratio, offering significant potential for improving drug-loading efficiency and controlled release, compared to traditional spheroid particles.

While simulating at experimental frequencies presents computational challenges, the trends observed in the simulations align well with the experimental results, validating the hybrid model’s accuracy. Future research will focus on refining computational methods to simulate at higher frequencies, and on exploring the impact of vibration parameters on the residual stress and mechanical properties of the silk fibroin particles. Additionally, the potential of these silk fibroin chips in drug delivery systems will be further investigated, including experimental studies on drug-loading efficiency and therapeutic performance.

This study represents a significant advancement in the processing of biocompatible materials, offering a novel and complementary approach for fabricating silk fibroin particles tailored for high-precision biomedical applications. By optimising vibration parameters in elliptical vibration micro-turning, this research provides a foundation for producing silk fibroin particles with precisely controlled dimensions and morphologies, paving the way for their effective use in advanced drug delivery systems.

## Figures and Tables

**Table 1:** Comparison of mechanical properties of steel, copper, Inconel, and silk fibroin [26,27,28,29,30].

Property	Steel	Copper	Inconel	Titanium Alloy	Silk Fibroin
Young’s modulus (GPa)	210	110	210	120	1–6.5
Hardness (HRC)	~60–70	~35–55	~40–55	~35–40	~2–3.5
Toughness (MPa·m^1/2^)	~50	~40	~30	~40	~0.5–1
Yield strength (MPa)	250–900	210–250	600–1000	900–1100	20–100

**Table 2:** Material parameters in orthogonal cutting model [28,29,30].

Material Parameters	Silk Fibroin	Diamond Tool
Density (g/cm^3^)	1.4	3.5
Young’s modulus (GPa)	5.2	1050
Poisson’s ratio	0.3	0.1
Static yield stress (MPa)	70	N/A
Tangent modulus (MPa)	172.4	N/A
Cowper–Symonds parameter *p*	7	N/A
Cowper–Symonds parameter *D* (s^−1^)	1140	N/A

**Table 3:** Processing parameters for machining trials.

Set	Cutting Mode	Vibration Frequency (Hz)	Vertical Vibration Amplitude (μm)	Nominal Depth of Cut (μm)
I	Conventional	N/A	N/A	1
II	Elliptical vibration	100	1	1
III	Elliptical vibration	500	1	1
IV	Elliptical vibration	500	1.5	1.5

## Data Availability

The data underpinning this publication is available from the University of Strathclyde KnowledgeBase.

## Nomenclature

FEM	Finite element method
FE-SPH	Finite element and smoothed particle hydrodynamics
SEM	Scanning electron microscope
SPH	Smoothed particle hydrodynamics
*α*	Tool clearance angle, °
*β*	Material hardening parameter
$\dot{\varepsilon }$ *_d_*	Dynamic strain rate, s^−1^
*ε_d_*	Dynamic strain
*π*	Mathematical constant, 3.1415926
*σ_d_*	Dynamic yield stress, MPa
*σ_s_*	Static yield strength, MPa
*a*	Horizontal vibration amplitude (cutting direction), µm
*b*	Vertical vibration amplitude (depth of cut direction), µm
*d_n_*	Nominal depth of cut, µm
*D*	Material parameter (Cowper–Symonds model)
*E*	Young’s modulus, GPa
*E_p_*	Plastic hardening modulus, GPa
*E_TAN_*	Tangent modulus, GPa
*f*	Vibration frequency, Hz
*p*	Material parameter (Cowper–Symonds model)
*R*	Chip radius of curvature, µm
*r_e_*	Tool edge radius, nm
*t*	Time, s
*V_w_*	Nominal cutting speed, m/s or mm/s
